# Immunity to SARS‐CoV‐2 induced by infection or vaccination

**DOI:** 10.1111/joim.13372

**Published:** 2021-08-05

**Authors:** Xaquin Castro Dopico, Sebastian Ols, Karin Loré, Gunilla B. Karlsson Hedestam

**Affiliations:** ^1^ Department of Microbiology, Tumor and Cell Biology Karolinska Institutet Stockholm Sweden; ^2^ Department of Medicine, Solna Karolinska Institutet and Karolinska University Hospital Stockholm Sweden

**Keywords:** antibody responses, B cells, COVID‐19, population immunity, SARS‐CoV‐2, vaccines

## Abstract

Adaptive immune responses play critical roles in viral clearance and protection against re‐infection, and SARS‐CoV‐2 is no exception. What is exceptional is the rapid characterization of the immune response to the virus performed by researchers during the first 20 months of the pandemic. This has given us a more detailed understanding of SARS‐CoV‐2 compared to many viruses that have been with us for a long time. Furthermore, effective COVID‐19 vaccines were developed in record time, and their rollout worldwide is already making a significant difference, although major challenges remain in terms of equal access. The pandemic has engaged scientists and the public alike, and terms such as *seroprevalence*, *neutralizing antibodies*, *antibody escape* and *vaccine certificates* have become familiar to a broad community. Here, we review key findings concerning B cell and antibody (Ab) responses to SARS‐CoV‐2, focusing on non‐severe cases and anti‐spike (S) Ab responses in particular, the latter being central to protective immunity induced by infection or vaccination. The emergence of viral variants that have acquired mutations in S acutely highlights the need for continued characterization of both emerging variants and Ab responses against these during the evolving pathogen‐immune system arms race.

## Graphical abstract

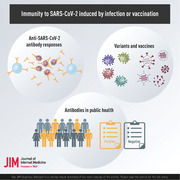

## Introduction

Antibodies (Abs) are produced by B cells in response to viral infection or vaccination. As key effector molecules capable of binding unprocessed antigen, they provide a first line of defense against subsequent exposures. In addition to the robust Ab responses produced by short‐lived plasma cells during an acute infection, lower levels of pathogen‐specific Abs are constitutively produced by long‐lived plasma cells in the bone marrow, providing serological memory for years after the pathogen has been cleared.

As secreted soluble proteins, the measurement of Abs in blood is amenable to scalable diagnostics aimed at determining responses to past infections and vaccines. Indeed, most viral infections and vaccines provide protection against re‐infection through the induction of neutralizing Abs that bind viral surface structures and block virus entry into target cells. During natural infection, CD8^+^ T cells play an important complementary role to contain the infection through their ability to eliminate already infected cells, while CD4^+^ helper T cells, amongst other functions, provide signals that support the development of Ab responses. Knowledge about quantitative and qualitative aspects of the Ab response to SARS‐CoV‐2, including durability and epitope specificities of the response is central to our understanding of anti‐viral immunity and offers information that can guide public health and clinical measures.

Since the virus emerged in late 2019, much effort has been directed to the characterization of innate and adaptive immune responses to SARS‐CoV‐2 with the aim to understand the roles of different immune functions in viral clearance. As in other viral infections, T and B cells work in concert alongside the instructive innate immune system to control SARS‐CoV‐2, with the adaptive arms displaying distinct response kinetics, mode of antigen recognition, effector functions and immunological memory, often consistent with textbook knowledge [1, 2]. As the vast majority of SARS‐CoV‐2 cases result in asymptomatic or mild disease (with elderly cases developing disease more frequently), our immune system generally responds appropriately, with diverse myeloid [3], lymphoid [4, 5] and non‐hematopoietic [6] lineages contributing to host defense and viral clearance. However, longer‐term consequences of COVID‐19, such as potentially auto‐reactive antibodies [7] and persistent fatigue in post‐acute COVID‐19 syndrome, or ‘Long Covid’ [8, 9], affect a subset of individuals, requiring further investigation.

While the seroprevalence resulting from natural infection is still too low to have a major impact on slowing the pandemic worldwide, ongoing viral spread and vaccine rollouts on a global scale will contribute to a reduced pool of susceptible individuals. However, SARS‐CoV‐2 has already displayed adaptation to its new host. This is evident from the identification of novel variants that continue to outcompete previous strains in many parts of the world [10, 11, 12], a process likely more widespread than appreciated that shows little sign of abating. Indeed, at the time of writing, the first European cases caused by the lambda variant of interest (C.37, first identified in Peru and with novel spike mutations with respect to variants of concern (VoC) [13]) were being identified, after the variant expanded rapidly in Latin America [14]. Furthermore, while current vaccines are highly effective in terms of protecting against severe disease and fatality, much less is known about their ability to blunt the transmission of different variants.

Here, we summarize current knowledge about the B cell and Ab response to SARS‐CoV‐2 with reference to key SARS‐CoV‐2 population serology studies and emerging research describing qualitative aspects of the Ab and memory B cell response. We also discuss vaccine‐induced immune responses and results emerging from the rapid worldwide roll‐out of vaccines against COVID‐19. Together, these themes are essential for appropriate COVID‐19 public health measures, epidemiological understanding and for informing molecular medicine.

## The B cell response to SARS‐COV‐2

**Fig. 1:**
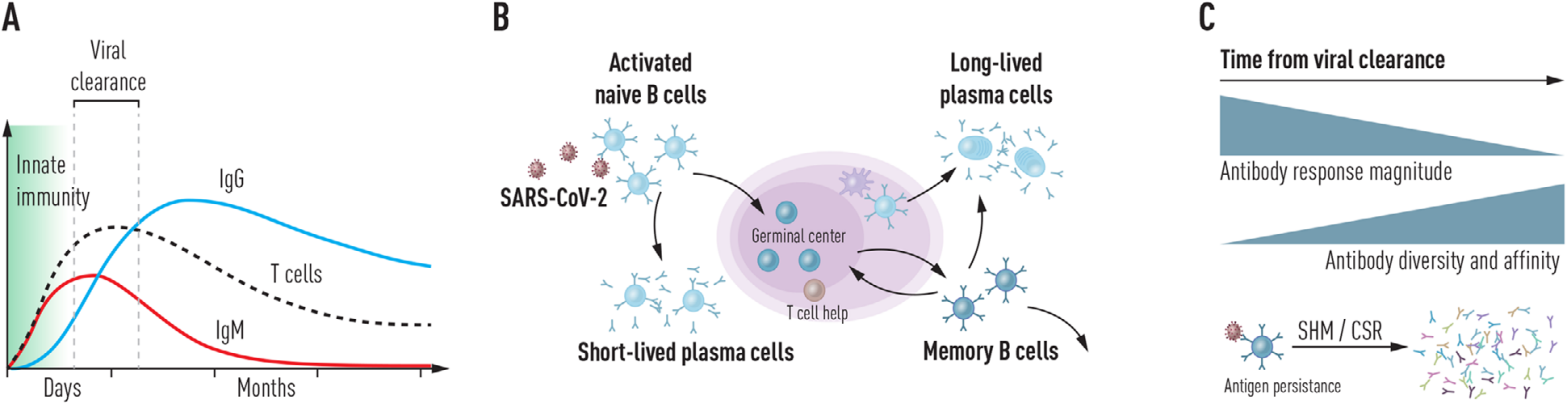
(a) Kinetics of innate and adaptive immune responses following SARS‐CoV‐2 infection. Following rapid action by the innate immune system immediately after virus exposure, B and T cell responses develop within the first weeks. Short‐lived IgM antibodies are produced by responding B cells initially and is followed by a more persistent and high‐affinity class‐switched response. (b) Production of highly diverse virus‐specific antibodies. After cognate antigen encounter, activated naïve B cells enter the germinal center (GC) where they receive T cell help to generate a high‐affinity antibody repertoire. Short‐lived peripheral plasma cells produce most antibodies during the infection, while GC‐derived memory B cells and bone marrow‐resident plasma cells cooperate to provide long‐lasting protection against re‐infection. (c) Maturation of the antibody response following viral clearance. While the magnitude of the antibody response gradually wanes after the virus replication is controlled, the quality of the B cell response continues to improve for several months following the infection

Our immune response to viral infections engages functions that combat the invading pathogen in a stepwise manner (Fig. 1). First, the innate immune system is engaged, which recognizes and eliminates foreign viral material and activates a signaling cascade that limits the spread of the virus to neighboring cells. The innate immune system may do a substantial part of the work to contain SARS‐CoV‐2 in some cases, such as asymptomatic or mild infections in children and young adults [15], although this is difficult to demonstrate empirically.

Shortly thereafter, T cells recognizing processed Ag presented by MHC class I and II molecules are recruited to destroy infected cells and orchestrate the immune response, while the IgM response develops in parallel. Multi‐valent anti‐viral IgM plays a prominent role during the early stages of infection, being the first isotype produced in response to the infection. Immunophenotyping of peripheral B cells during SARS‐CoV‐2 convalescence has shown that un‐switched IgM^+^ memory B cells, as well as classical, switched B cells form part of the circulating memory population and can remain stable for months [16]. Memory T cells reactive to a diverse set of SARS‐CoV‐2 epitopes are detectable in convalescent individuals [17, 18]. However, cross‐reactive T cells were shown to be present in a high percentage of pre‐pandemic and seronegative individuals who were unexposed to SARS‐ CoV‐2 [17, 18, 19], consistent with a more promiscuous nature of T cells, compared to B cells [20, 21, 22]. Reports suggest that cross‐reactive IgM memory B cells elicited by previous exposures to endemic coronaviruses (CoVs) may be engaged in the response to SARS‐CoV‐2 in a minority group [23, 24, 25], while the IgG response, especially to the spike glycoprotein, shows a high level of specificity for SARS‐CoV‐2. Whether prior responses to endemic CoVs offer some level of protection against clinical COVID‐19 requires further exploration and will be discussed again later.

De novo B cell responses to SARS CoV‐2 are critical for generating effective neutralizing Abs against the virus. Following the initial IgM response, which wanes relatively quickly with viral clearance [26], class‐switched Abs are generated, primarily IgG and IgA. After acute infections, IgG titers remain elevated and relatively stable for many months or years. How rapidly the response declines, sometimes beyond detection, depends on the magnitude of the peak response, the subtypes of antibodies involved, and the relative contribution of short‐lived and long‐lived plasma cells to the circulating IgG levels. In the case of reinfection, antigen‐specific memory B cells are quickly engaged improving immunological efficiency. It follows that repeated antigenic stimulation bolsters immunological protection; however, as mutations in key Ab epitopes in the S glycoprotein come into play, with different virus variants arising, the level of protective immunity may be compromised. Therefore, the degree to which immunity induced by previous exposure to one strain protect against others, over longer timescales, remains to be determined. Future studies of fate decisions of B cells after infection and vaccination, alongside their detailed molecular characterization, will further our understanding of factors influencing the nature, potency and longevity of B cell memory. Furthermore, as T cells comprise the other major arm of the adaptive immune response (for SARS‐CoV‐2 excellently reviewed in [27, 28]), studies delineating the cooperation between T and B cells are advantageous to fully understand the control of SARS‐CoV‐2 [29].

### Serology

As the SARS CoV‐2 S glycoprotein harbors the well‐exposed receptor‐binding domain (RBD), an immunodominant determinant of the trimeric surface spike glycoprotein, responses against S or RBD is the best indicators of past infection for individual and population studies. A strong support that almost all SARS CoV‐2 infections generate class‐switched antibody responses comes from population studies from Iceland (*n =* >30,000) [30], where more than 90% of PCR‐positive persons surveyed developed anti‐S IgG. An elegant smaller study (*n* = 963) from Germany [31] arrived at 94.4%, while we and others have shown in smaller cohorts [32, 33] (e.g., care home residents [34]) that the majority of confirmed PCR‐positive persons seroconvert, even in mild cases [35, 36, 37], although Ab titers vary widely, with a subset of low titer responses scoring close to the detection limit of the assay. Further studies are needed to more accurately quantitate low titer antibody responses (e.g., with microfluidic serology [38]) to understand if there are cases where Abs do not develop, and why. The time to seroconversion has been found to vary by several weeks between recently infected individuals [39] (average time for IgG development: 10 days post‐PCR; slower in less severe cases), which reflects the time required for class‐switching and the generation of sufficient concentrations of IgG in the circulation for detection. It appears that IgG_1_ isotypes predominate over IgG_3_ after SARS‐CoV‐2 infection [40].

Although a couple of early studies indicated defective T_fh_ and GC function [41], or loss of memory B cells after severe infection [42], this is not the typical immune response to the infection [29, 30, 43]. Since these early reports, several studies have shown that antibodies encoded by SARS‐CoV‐2‐specific memory B cells undergo affinity maturation with somatic hypermutation levels similar to those achieved in response to other acute viral infections [44, 45], while memory T cells also display comparable functional longevity [46, 47], in‐line with clinically mild disease in the majority infections.

The Ab response to SARS‐CoV‐2 in adults and children [48] appears to be generally robust, although it is clear that the magnitude of the serological peak response differs greatly, at least 1000‐folds, between seroconverters [49, 50, 51, 52, 53], which is generally associated with differences in individual COVID‐19 severity manifestations. Generally, as COVID‐19 worsens clinically and the inflammatory response progresses, the more Abs are produced—with the highest titer neutralizing responses observed in patients receiving more intensive care over protracted disease courses [49, 54]. Similar observations were made in the context of SARS‐CoV [55] and MERS‐CoV [56, 57] and are consistent with prolonged/enhanced viral replication signaling increased Ab production. In contrast, in asymptomatic or mild COVID‐19 cases, viral replication is controlled sooner after exposure by a combination of innate and adaptive immune functions [58]. Notably, children have been found to generate lower Ab titers compared to adults post‐infection [48], which likely reflects less severe pathology in the young. However, it should be noted that mild infections can generate Ab responses at titers comparable to those seen during severe disease, underscoring the high variation in the magnitude of the response between individuals. Genetic [59], clinical [60], environmental [61] and stochastic factors influence the magnitude of the humoral response to different pathogens, but studies that investigate the relative contribution of these at individual and population levels are generally lacking. A new pandemic virus sweeping through the human population offers valuable opportunities to study how de novo immune responses develop in populations and how these differ between age groups and sub‐populations, especially as *big data* analysis truly takes the stage.

Antibody class‐switching to IgA seems to be particularly dependent on the clinical picture, with more severe COVID‐19 cases developing higher titers to protect mucosal surfaces, such as in the gastrointestinal and upper respiratory tracts [50, 62], while asymptomatic/mild cases with less disseminated pathology do not always engender detectable anti‐viral IgA in peripheral circulation. Notably, recent research has shown that IgA titers can persist for several months after a negative PCR test and alongside viral antigens in the GI tract [63]. The entire length of the intestine of rhesus macaques (a valuable translational model for SARS‐CoV‐2) has been found to harbor viral antigen at 3 days post‐infection [64], and the gastrointestinal tract is known to harbor a large number of IgA‐secreting plasma cells. As ACE2, the receptor for SARS‐CoV‐2, is widely expressed in the human small intestine, parasympathetic ganglia and some other compartments [65]—and neurological phenotypes have been documented for a subset of SARS‐CoV‐2 infections [66, 67]—additional studies of these sites using primary tissue are needed to determine viral tropism, pathophysiological mechanisms and the nature of the B and T cell response in situ. Indeed, the past decade has seen important advances in our understanding of tissue‐specific immune mechanisms, including those of B cells at serosal surfaces [68, 69, 70], that often implicate cellular and organism‐wide metabolism dysregulation, noting that a worse SARS‐CoV‐2 prognosis (like responses to different infections [71] and vaccinations [72, 73]) is associated factors predisposing to and arising from obesity.

The quality of the Ab response is also influenced by age [74], as is the case for T cell responses. Given the increasing risk of severe COVID‐19 in the elderly, age‐dependent immunological mechanisms are particularly important to elucidate, as these could be targeted to improve responses to natural infection and vaccination. For example, a recent study mining historical (pre‐pandemic) antibody repertoires for anti‐SARS‐CoV‐2 reactive clones found several germline‐like clones to be present in unexposed individuals, although these became less frequent in persons aged 60 and over, who had a more restricted antibody repertoires [75, 76]. Another study reported SARS‐CoV‐2‐specific memory B cells with weak cross‐reactivity to other coronaviruses to be more prevalent in pediatric samples than in adults [77], while cross‐reactive serum Abs have also been found to be present to a greater extent in children compared to adults [24]. Unfortunately, only a limited number of studies have yet analyzed cellular and antibody responses in school children and infants. Early results indicate that pediatric cases, like adults, develop class‐switched and neutralizing antibody responses [48, 78, 79, 80, 81]. Although studies in such cohorts are complicated for several reasons, pediatric cohorts are essential for understanding disease biology [82], viral transmission and shaping public health approaches to education.

As regards cross‐reactive Ab responses, it has been reported that a small proportion of individuals recently infected with endemic (seasonal) coronaviruses (CoVs), such as OC43, 229E, NL63 and HKU1 (or SARS‐CoV), show anti‐SARS‐CoV‐2 S reactivity and neutralizing ability at the polyclonal serum level, indicating that some anti‐S binding modes may be cross‐reactive between related CoVs [24]. Another study reported convergent clonotypes between SARS‐CoV and SARS‐CoV‐2 [83], and relatedly, a back‐boost of antibodies specific to seasonal CoVs was reported in individuals recently infected with SARS‐CoV [84] and SARS‐CoV‐2 [25, 85]. This supports that different SARS‐CoV‐2 S epitopes can trigger memory B cells generated by related viruses in a subset of individuals previously infected, although the extent to which they (and Ab cross‐reactivity between alpha and beta coronaviruses [86, 87]) shape population‐level outcomes remains to be established. In one study, such cross‐reactive Abs were reported not contributing to the neutralizing Ab response [88].

### Antibody specificity

Elegant studies have shown that the response to the SARS‐CoV‐2 S glycoprotein is highly polyclonal using a broad spectrum of immunoglobulin heavy chain variable (IGHV) genes [88, 89, 90], with many lineages found to display potent neutralizing activity [63, 91, 92, 93]. An informative Ab repertoire study over the disease course revealed that in the early, acute response, B cells with a limited set of V genes are recruited to fight the infection, presumably the most potent germline configurations within an individual, before progressing to a highly polyclonal response with broader V gene usage during the first week of infection [44], as additional naïve B cells are recruited into the activated pool. Clonal expansions have been detected approximately 2 weeks after the onset of symptoms [94].

Studies of S‐reactive neutralizing Abs have shown convergence at the level of immunoglobulin germline V, D and J gene usage between unrelated individuals [44, 92, 93, 95], suggesting that some Ab configurations are preferred and do not need to undergo affinity maturation to contribute to the early response; consistent with endemic CoVs shaping, the mammalian immune response over evolutionary time. The exposed nature of the RBD in the context of the S trimers on the viral surface, and the fact that potent neutralizing antibodies are readily isolated from convalescent individuals soon after they have cleared the infection, suggest that SARS‐CoV‐2 is a neutralization‐sensitive virus. While most neutralizing anti‐S antibodies are directed against the RBD, the N‐terminal domain (NTD) is also a target for neutralizing antibodies [96]. Additional neutralizing targets, such as in the S2 region are of interest since they may have cross‐neutralizing capacity and are less sensitive to emerging mutations and/or deletions in the RBD and NTD [88, 97].

Structural studies of SARS‐CoV‐2‐directed neutralizing antibodies show that Abs bind the RBD according to one of several modes. Class I antibodies bind the RBD in the ‘up’ conformation, while Class II antibodies bind the RBD in both the ‘up’ and ‘down’ conformations [98]—both classes target an epitope region that overlaps with the RBD‐ACE2 interface. Additional classes of RBD‐directed neutralizing Abs bind with different angles of approach, some of which do not appear to overlap the ACE2 binding region [99]. In the cases where structural analysis was performed, it was shown that most of the neutralizing monoclonals identified from previously infected persons use both heavy and light chain residues to contact the epitopes of the RBD. Thus, further examination of both heavy and light chain V(D)J gene usage in SARS‐CoV‐2 neutralizing Abs is of interest to understand potential reasons for inter‐individual differences in the response, which in part is stochastic, but which may also be influenced by genetic factors since immunoglobulin genes display considerable allelic diversity in the population.

As an ever‐greater number of SARS‐CoV‐2 S‐specific monoclonal Abs are isolated, and individual Ab repertoires are deep‐sequenced, it is becoming increasingly clear that Ab responses to S sample a broad range of V(D)J arrangements; however, some genetic features stand out. Studies using RBD‐probes to sort antigen‐specific cells to isolate neutralizing mAbs have demonstrated that Abs using IGHV3‐53, and the related IGHV3‐66 gene, are frequently used in Class I neutralizing Abs. These two genes both encode an NY motif in the HCDR1 and an SGGS motif in HCDR2 region of the Ab that interacts with the target epitope [100]. In most cases, this binding mode requires that the Abs have a short HCDR3 region. Class II neutralizing antibodies also display preferential IGHV3‐53 gene usage, but with less dependence on HCDR3 length. Furthermore, ultrapotent RBD‐directed neutralizing IGHV1‐58‐using antibodies were isolated from infected individuals [101]. A recent structural study of such antibodies showed their capacity to bind in an epitope region that is not affected by several key mutations present in VOCs [102], illustrating the benefit of profiling plasma neutralizing responses against different viral variants to isolate potent and broadly neutralizing antibodies.

While preferential IGHV gene usage was reported in early studies of RBD‐specific Abs, the response to the full trimeric S protein is quite diverse and engages many different IGHV genes, including those that are also highly used in many other viral infections and in the IgG repertoire in general, such as IGHV1‐69, IGHV3‐23, IGHV3‐30 and IGHV3‐30‐3 [88, 103, 104, 105]. Additional genetic features are likely to be revealed as more infections and vaccinations are characterized in different populations. The Coronavirus‐binding Antibody Sequences and Structures Database (http://opig.stats.ox.ac.uk/webapps/covabdab/) has emerged as a useful resource where scientists can deposit and search Ab sequences to identify features of anti‐SARS‐CoV‐2 humoral immunity. Another useful resource is the Coronavirus Immunotherapy Consortium (https://covic.lji.org), a global partnership to accelerate Ab‐based therapeutics against SARS‐CoV‐2.

Outside the immunoglobulin loci, human genetic studies will further our understanding of disease susceptibility and immune responses in different cohorts. For example, common single‐nucleotide polymorphisms around important B cell genes, such as *TYK2* [106], increase the risk of severe COVID‐19 [107], as can antibody immunodeficiencies of diverse etiologies [108, 109, 110]. For example, individuals with common variable immunodeficiency (CVID, for example, associated with deletions in *CD19*, *MS4A1* (CD20) and *CR2* (CD21) that impair B cell function) have been found to be at increased risk of severe disease [111] and require further study. Furthermore, immunosuppressive medication can also interfere with antibody seroconversion and reduce viral clearance [112], and convalescent plasma therapy has shown to be efficient in B cell‐deficient patients with COVID‐19 [113].

## Durability of immunity after natural infection

How long immunological protection lasts after SARS‐CoV‐2 infection is a critical metric that strongly impinges upon how the pandemic will play out, epidemiologically and in terms of public policy, since immune protection is sensitive to the emergence of viral immune escape variants. Moreover, many previously infected individuals would like to know their risk of acquiring COVID‐19 a second time, and others would like to know how long the protective effect of their vaccination will last. Past‐controlled human infection experiments with seasonal CoVs have demonstrated that infection‐induced antibodies correlate with protection to re‐challenge [114, 115, 116, 117, 118], although re‐infection was possible. Indeed, it is important to appreciate that immunity represents a spectrum of protection that is dependent upon numerous cell types (e.g., B and T cells, NK cells [119, 120] and macrophages [121]) physical barriers that are more or less effective in different individuals, along with differences in environmental influences (e.g., medications with side‐effects on the immune system [122]), general health status and age. Therefore, it is more useful to consider infection‐induced immunity as a measure of reduced risk upon re‐infection, similar to that induced by current SARS‐CoV‐2 vaccines that are highly effective at preventing severe COVID‐19, but not necessarily viral transmission. It is hoped that the coming years will yield important advancements in quantitative immune profiling at population levels so that the relative contribution of different protective barriers and environmental factors can be estimated [123].

Although several studies have now shown that anti‐SARS‐CoV‐2 Ab titers decline from peak levels with time [124, 125, 126], as Ab titers do for all cleared infections, neutralizing Ab responses and virus‐specific memory B cells quantitated by flow cytometry were described to remain prevalent in the peripheral circulation up to 8 months or more post‐infection—with most previously infected individuals still harboring good levels of circulating antibodies at this time point. One large study showed 13% of individuals lost detectable IgG titers 10 months post‐infection [127]. How this will play out in other cohorts, in the longer‐term and after vaccination, remains to be seen. Some of the best empirical evidence for Ab‐mediated protection from COVID‐19 comes from a study of *n* = 12,541 healthcare workers in the United Kingdom, which reported a substantially reduced rate of re‐infection over 6 months in persons previously antibody positive: for example, no symptomatic cases were detected in persons previously antibody positive to S or N in the study period [128]. Smaller studies [129] in different contexts (e.g., a fishing vessel outbreak [130] and elderly care homes [131]) have also illustrated the highly protective effect of serum antibodies.

Evidence suggests that Ab immunity to endemic seasonal CoVs and SARS‐CoV wanes within a 2‐ to 3‐year period in the majority of those previously infected [79, 132, 133, 134, 135], which represents a combination of lack of cross‐protective immunity to mutating seasonal strains, rhythms in the circulation of different CoVs [136, 137, 138], and loss of antibody‐producing cells over time with aging [139]. Several case studies of SARS‐CoV‐2 reinfection have been documented in the literature [140, 141], although it is not known at present whether these were caused by infection with a different strain, waning immunity or unique clinical features explained the occurrence. Indeed, early results indicate that the potency of engendered antibodies is generally reduced when faced with a new S variant strain [88, 142, 143, 144], suggesting less effective but not abolished immunity due to changes in neutralizing antibody epitopes in S. This is similar to what has been observed for vaccines based on the original Wuhan, China, strain [145, 146, 147, 148]. Neutralizing antibody titers after mild SARS‐CoV‐2 infection were reported to be comparable to those engendered by first‐generation COVID‐19 vaccines [149, 150].

### Vaccine‐induced immunity and protection

The correlate of protection for almost all clinically licensed vaccines is neutralizing Abs [151], and emerging data suggest that the current SARS‐CoV‐2 vaccines are no exception [152]. As discussed above, evidence that the presence of neutralizing Abs is associated with protection against SARS‐CoV‐2 in humans was illustrated early in non‐human primates receiving the neutralizing monoclonal Ab LY‐CoV555 (Bamlanivimab) before a high‐dose SARS‐CoV‐2 exposure, which conferred protection [153], while adoptive transfer of purified IgG from convalescent non‐human primates protected naive animals against challenge with SARS‐CoV‐2 [154].

**Fig. 2:**
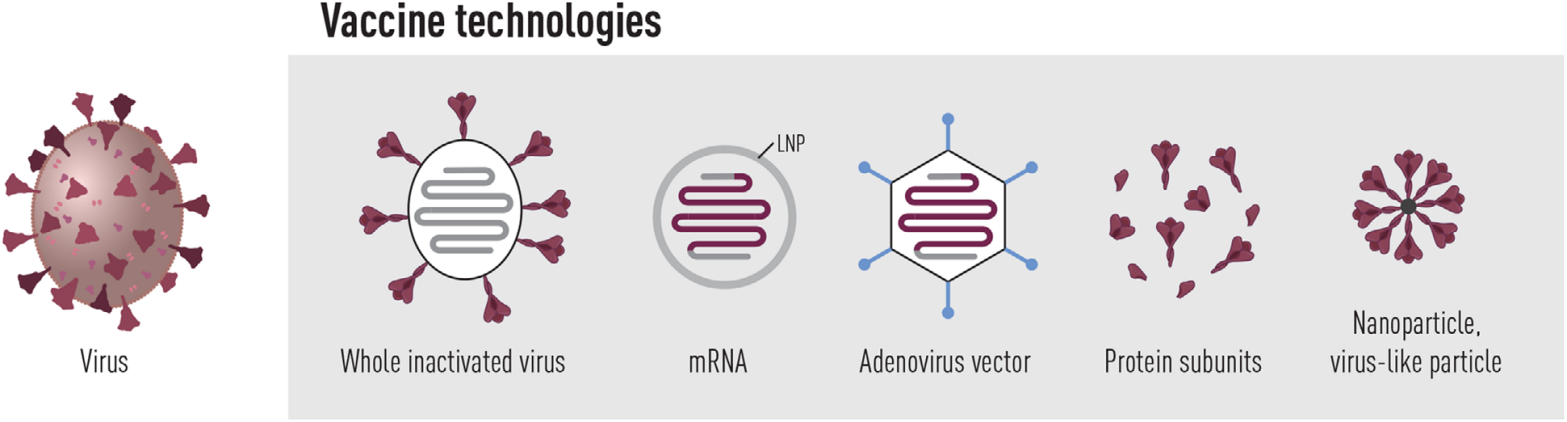
Vaccine platforms in use or under clinical evaluation to prevent COVID‐19. Currently, several inactivated whole virus vaccines as well as SARS‐CoV‐2 spike‐encoding vaccines based on mRNA or adenovirus technology are approved for clinical use. Furthermore, spike protein subunit vaccines have shown high efficacy in phase 3 clinical trials and should be approved for use shortly

Fortunately, SARS‐CoV‐2 is relatively easy to neutralize and even though variants that partly escape Ab responses have already evolved, the human neutralizing Ab response is quite effective at blocking infection if the antibody titers are of sufficient magnitude. As the S protein on the surface of SARS‐CoV‐2 is the target for neutralizing Abs, all currently licensed vaccines are based on this antigen. Once the original pandemic strain was identified, several vaccine companies initiated the development of different vaccine platforms with the goal to elicit anti‐S Ab responses and protective immunity (Fig. 2). The historical approach of producing vaccines, based on whole inactivated virus, was also used for SARS‐CoV‐2 vaccines, and several such vaccines are now approved for clinical use [155, 156]. However, the COVID‐19 vaccines that have so far shown the best efficacy, and are most broadly distributed globally, are based on modern molecular biology‐based technologies, in particular mRNA‐based vaccines encoding the S protein [149, 157, 158]. Several genetically modified replication‐incompetent adenovirus vectors encoding S are also approved [159, 160]. Furthermore, after highly successful clinical trials typified by strong anti‐SARS‐CoV‐2 neutralizing antibody responses [148, 161, 162], vaccines based on recombinant S protein, in some cases produced as nanoparticle‐like structures, given with adjuvant, are also expected to soon be approved for clinical use.

A common misperception is that the SARS‐CoV‐2 vaccine technologies are new concepts of which there is limited prior knowledge. However, the mRNA and adenovirus vector platforms have been studied already for a few decades in the development of vaccines against other pathogens, and multiple vaccine candidates based on these technologies have been evaluated in clinical trials [163, 164]. In addition, the human papilloma virus (HPV) and hepatitis B vaccines, which have been successfully used in humans for many years, are examples of subunit vaccines that are based on modern recombinant protein technology. Being formulated for intra‐muscular injection, these vaccines provoke mainly IgG responses, although lower levels of IgM and IgA are induced by mRNA vaccination [165], the degree to which may be influenced by host factors. From HPV vaccination, also delivered intra‐muscularly, it is known that vaccine‐induced IgG distributes to mucosal surfaces where it provides a high degree of protection against cervical cancer [166]. COVID‐19 vaccines given intra‐nasally are under development [167], and future work will reveal if such vaccines have advantages, for example, for better curtailing transmission if a higher degree of neutralizing IgA antibodies are present within the respiratory tract [168].

Importantly, mRNA, adenovirus vector and recombinant protein vaccines are molecularly well‐defined vaccines that are produced with a high degree of precision and reproducibility. The scale of the COVID‐19 pandemic puts unprecedented demands on vaccine developers, production facilities and local infrastructure to administer vaccinations—requiring greater investment in public health to counter future threats. It also requires international cooperation to successfully distribute the vaccines to all countries of the world to control the pandemic. While equitable access is still far from a reality, mechanisms that are beginning to address such issues are now in operation (https://www.who.int/initiatives/act‐accelerator/covax) and should be prioritized in future fights against pandemics.

### Efficacy of SARS‐CoV‐2 vaccination

The current SARS‐CoV‐2 vaccines were approved after multiple successful clinical vaccine trials demonstrating high efficacy. Efficacy is determined as the percentage reduction of cases with symptomatic COVID‐19 in those who were vaccinated compared with the number of cases observed in a placebo control group. It is not trivial to assess differences in efficacy between vaccine platforms or even vaccine products using similar platforms, because trials are performed at different locations, during different time periods, and sometimes, with different endpoints or criteria for scoring positive cases. However, in essence all approved vaccines have shown very good efficacy, demonstrating induction of Ab levels of similar or higher magnitude as those observed in convalescent individuals, and near‐complete protection against hospitalization and severe disease [152, 169]. As a comparison, vaccine efficacy for seasonal influenza is typically 30%–60%, depending on the year, and how well the vaccine matches the circulating virus strains.

In addition to the clinical phase 3 studies, very encouraging ‘real world’ data are now becoming available. Reports from the early initiated mass vaccination using the Pfizer/BioNTech's mRNA vaccine in Israel demonstrated 46%–74% efficacy after the first dose and 87%–95% after the second dose in terms of protection against symptomatic disease [170, 171, 172]. Another large study of healthcare workers in the UK (in which participants were PCR tested every second week) showed vaccine efficacy for protection against symptomatic infection to be 70% after the first dose and 85% 1 week after the second dose of the Pfizer/BioNTech's mRNA vaccine [173]. In a report issued by the UK government, the first dose was 78% and 75% effective at preventing hospitalizations after alpha or delta virus infection [174], while two doses conferred 92% and 94% protection, respectively. Future studies will also inform the duration of vaccine‐induced protection. Interestingly, work from Israel suggests that mass‐vaccination not only protected the vaccinated individuals, but also provided cross‐protection to unvaccinated contacts, such as children under the age of 16 [175]. Recent studies showed that the largest benefit of vaccination was obtained after two vaccine doses [176].

Additional reports from vaccine follow‐up studies in different countries are likely forthcoming. It will be important to determine potential differences in vaccine efficacy between different age groups for different vaccines, if re‐infections are more frequent in the elderly and, importantly, whether the frequency of breakthrough infections increases over time as vaccine immunity wanes and/or new variants spread. Furthermore, a deeper understanding of adverse vaccine‐induced responses, as reported for all platforms in use, is necessary to gain the public's trust.

### Protection against VoCs

**Fig. 3:**
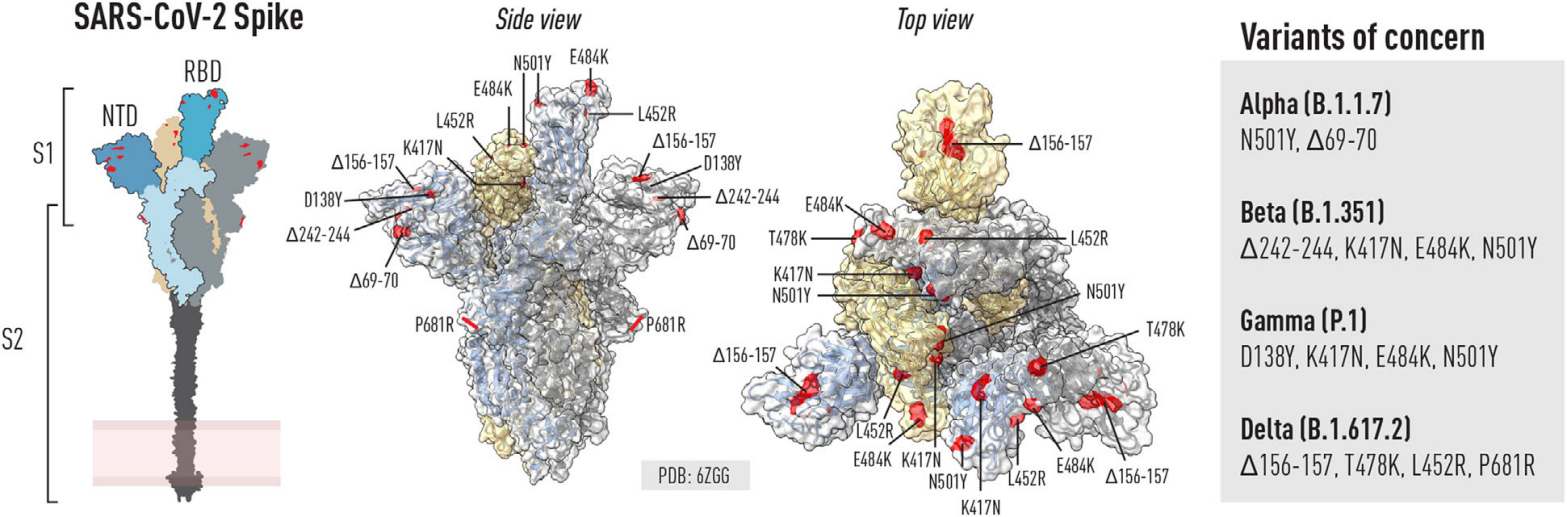
Schematic of the SARS‐COV‐2 spike glycoprotein and location of mutations in VoCs. Left: Schematic of the spike trimer highlighting key domains including the RBD, NTD and S2. Right (side and top view): ChimeraX was used to illustrate selected residues in the spike trimer that are mutated in selected VOCs known at the time of writing. Mutations can be common or unique to different VOCs

A major concern is that the vaccine efficacy will be reduced with the spread of viruses that carry mutations in key neutralizing Ab epitopes, allowing the virus to partially evade Ab recognition. If so, the vaccines may need to be updated to induce potent neutralizing Ab responses against new strains—analogous to seasonal flu vaccinations. Recently, it was documented that neutralizing Ab responses induced by the current vaccines [177, 178], which are based on the original Wuhan SARS‐CoV‐2 spike, are less active against the rapidly‐spreading delta variant (B.1.617.2, originally isolated in India) [179]. Similar loss of neutralization potency in vaccinated sera was reported against alpha (B.1.1.7, originally isolated in the UK), beta (B.1.351, originally isolated in South Africa) and gamma (P.1, originally isolated in Brazil) variants [10, 180, 181, 182, 183, 184] (Fig. 3). Consistent with this, emerging data from the Israeli Health Ministry [185] suggest that the current circulation of the delta variant there has already resulted in a reduction of vaccine efficacy in preventing symptomatic disease from above 90% to 64%, and this may drop further as individual antibody titers wane—suggesting the need for third doses before the winter season to protect the most vulnerable groups.

Despite decreased efficacy, the current vaccines clearly reduce the number of cases developing severe disease and hospitalization also in regions where these variants dominate [186]. Given the high global transmission levels and the risk of new variants arising, continued virus surveillance will be needed for a foreseeable future. This is especially important as vaccine‐induced Ab levels wane over time, in some cases below protective levels. So far, studies of breakthrough infections in vaccinated persons demonstrate that these are predominantly caused by VoCs [12, 187]. Therefore, boosting the immune response with a variant vaccine may be needed as soon as this falls, especially in the elderly population. However, it is important to note that, even if some classes of Abs lose reactivity, the overall anti‐S Ab response, including to the RBD, is polyclonal and consists of Abs that also recognize the VoCs. Although virus‐specific T cells are less affected by the mutations in the VoCs [188], it is known for many decades that cross‐strain‐reactive T cells recognizing conserved epitopes of influenza virus are not sufficient to protect against reinfection and reduce disease burden. This is evident from the fact that updated seasonal influenza vaccines are required yearly to elicit Abs to the new circulating strains [189]. The role of neutralizing Abs as a correlate of protection for COVID‐19 vaccines is becoming increasingly clear, as recently discussed [190].

An interesting study demonstrated that Abs elicited in individuals infected with the SARS‐CoV‐2 beta variant (B.1.351) displayed potent cross‐neutralizing activity against both the original Wuhan virus and the alpha variant (B.1.117) [191]. This is promising for future vaccine strategies based on variant S antigens. Spike subunit vaccines, especially mRNA‐based vaccines, can be rapidly redesigned and produced to match variant viruses and can be administered several times, unlike adenovirus‐based vaccines that are hampered by anti‐vector immune responses. Furthermore, sequential immunizations combining different vaccine platforms is a promising approach under evaluation in clinical trials and already in practice in some places. Heterologous prime‐boost regimens show promising results, consistent with results from previous pre‐clinical research using such regimens to stimulate immune responses against other pathogens.

### Induction and durability of vaccine‐induced immunity

While one dose of most of the currently approved COVID‐19 vaccines is sufficient to provide protection against moderate or severe disease, most SARS‐CoV‐2 vaccines require two doses to achieve full and durable protection. The higher Ab titers obtained after the second vaccine injection also provide improved cross‐neutralizing activity against VoCs [182]. A strategy in situations of vaccine shortage is to vaccinate as many persons in the population as possible with one dose and delay the second dose to provide some level of protection to as many as possible to reduce the number of severe cases and hospitalizations at large. The drawback of this approach is that optimal protection is not achieved in the period between the first and the second dose, resulting in a group of people who may be susceptible to reinfection, especially with VoCs such as delta, and which may contribute to the transmission chain. How the vaccines are administered in the real world is influenced by practical considerations such as availability of vaccines and estimations about what vaccination strategies will most effectively curb high transmission rates in the population.

As for SARS‐CoV‐2 and other infections, vaccine‐induced Abs naturally wane with time. However, as discussed above, memory B cells remain and rapidly expand and differentiate into Ab‐secreting plasma cells [192] upon re‐exposure. Ongoing and future studies will reveal how durable the vaccine responses to the SARS‐CoV‐2 vaccines are. Since the level of Abs (correlate of protection) required to prevent infection or disease is still not defined, the optimal time point for additional booster vaccination is not yet known and may differ between different age groups. Studies have shown that mRNA vaccine‐induced Abs were detected more than 6 months after vaccination [193]. However, at this time the Ab levels had waned to levels where a significant reduction in the cross‐neutralizing capacity to variant strains was observed [194]. It is therefore critical to monitor the evolution of new variants, determine how well they are neutralized by the original vaccine‐induced Abs and plan for booster immunizations with variant‐updated vaccines accordingly.

### Vaccination after recovering from COVID‐19

Protective immunity after SARS‐CoV‐2 infection is generally good, and the risk to be re‐infected is low although not non‐existent [128]. The extent to which protective immune responses last depends upon the time that has elapsed since the infection and how robust the peak Ab responses were. Other factors such as health status, age, and whether the re‐exposure is with a VoC to which the pre‐existing Abs may be less effective also play a role [195]. Ab responses to vaccination in individuals who were previously infected with SARS‐CoV‐2 are potent, at the same or higher level than those achieved with two vaccine doses [196, 197, 198]. Some countries have therefore recommended that individuals with a documented prior SARS‐CoV‐2 infection only need one vaccine dose, and/or should wait an extended period before receiving the second dose. Antibodies induced by vaccination, to a large extent, appear to resemble those induced by natural infection. Clonal lineages elicited by a prior infection can be expanded and improved upon by subsequent vaccination. Consequently, the neutralizing Ab activity is broader and more cross‐reactive against variant viruses in such cases [199]. Preliminary data suggest vaccine‐elicited Abs are more focused on the RBD on the S protein than Abs elicited by infection [200]. Further studies aimed at dissecting the Ab response following natural infection or vaccination are needed to determine if there are qualitative differences in the breadth of the neutralizing Ab response between individuals, including against VoCs.

The pandemic has resulted in that new vaccines were successfully developed in record time, capitalizing upon years of scientific advances in molecular technologies and in‐depth understanding of immune and infection mechanisms. In the future, it is possible that annual vaccination against SARS‐CoV‐2, analogous to seasonal influenza vaccines, will be recommended for some age groups, depending on disease susceptibility and the magnitude of community transmission. Furthermore, it will be important to address in large future studies, what Ab titers are required for protection against disease or infection.

## Antibodies, immunity and public health

Antibody testing remains our best way to estimate past SARS‐CoV‐2 infection and a positive vaccine response, although many factors, such as waning responses, need to be considered. In the context of SARS‐CoV‐2, anti‐S Abs are particularly important, as they develop in the majority of infections [30]. As IgM and IgA isotypes generally wane in the peripheral circulation with viral clearance, they are not as useful for monitoring individual and population responses as IgG molecules, although they may help illuminate the clinical picture in a COVID‐19 patient. However, not all Ab tests are of equal sensitivity and specificity [201, 202, 203, 204, 205, 206], and while high‐quality tests are now available, there is a range of tests of varying performance. The considerable inter‐individual differences in anti‐viral Ab levels and nature [33, 39, 48] call for international guidelines and improved regulatory standards for Ab testing. This would facilitate comparisons between studies monitoring previous infection and vaccination, and positively impact clinical medicine related to COVID‐19 at the individual level.

Currently, mass‐produced tests for clinical and community use (many of which have low specificity and sensitivity) are based on individual domains of S (e.g., S1, S2, RBD) as these are easier to produce for large distribution. However, as some of these antigens are not produced in a native form, some antigenic specificities may be missed, especially when Ab titers are in the low range, for example, with time from infection/vaccination and if the assay platform has a relatively high limit of detection. For example, lateral flow tests (ideally suited for quick results in the field and for large population surveys) that require a drop of fresh blood to be deposited on a membrane, struggle to detect low titer responses, while anti‐SARS‐CoV‐2 IgG can be detectable at a 1:200,000 serum dilution by ELISA [33]. Therefore, the quality of population seroprevalence studies may vary greatly depending on the configuration of the test that was used.

Even different versions of stabilized S glycoprotein trimers, which aim to mimic the native S conformation in vivo, have different antigenic properties that can impart important differences to detailed molecular studies of Ab specificities, which could be important when analyzing VOCs. The choice of Ag is also important for biological, rather than technical reasons. For example, approximately 10% of PCR‐positive individuals do not have detectable anti‐nucleocapsid (N)‐directed IgG responses [33, 36, 48], although they do have anti‐S and anti‐RBD responses at the same time. In the future, platforms with low limits of detection and using novel statistical methods will improve upon antibody test sensitivity, specificity and error rate [38], while new technologies for home‐based tests will facilitate the logistics of population‐wide testing as routine. It seems reasonable that public health authorities provide additional guidelines to public healthcare systems to help them navigate a large number of products on the market, especially as assays for different SARS‐CoV‐2 variants enter the market.

Despite these technical limitations, thanks to sero‐surveillance, before vaccines were widely distributed, we know that no location worldwide had achieved herd immunity after natural SARS‐CoV‐2 infection, except perhaps isolated populations or clusters. For example, Manaus, Brazil suffered amongst the highest seroprevalence in response to the first wave of SARS‐CoV‐2, estimated at greater than two‐thirds of the population [207], yet this population was not spared a severe second wave and ongoing infection burden. Other locations with a high seroprevalence after the first wave included New York City, USA (20%) [208] and Lombardy, Italy (23%) [209]. Seroprevalence has been shown to increase alongside ongoing viral transmission [210], and the toll on public health has been severe.

Evaluation of SARS‐CoV‐2 spike‐specific IgG responses at the population level is, therefore, critical for determining public health measures that aim to curtail transmission. Such analyses need to be increasingly applied to different demographic groups, including children, to gain further epidemiological understanding and COVID‐19 management strategies. Monitoring individual‐ and population‐level Ab responses after vaccination will also be critical for determining the efficacy of different vaccine platforms, including how long Ab responses last and for issuing ‘vaccine/past infection certificates’, which will be required for several lines of employment (e.g., elderly care home staff), much like the Hepatitis B vaccination is required to practice human and veterinary medicine in most developed economies. Indeed, it could be argued that antibody test results are more useful for demonstrating immunity in those previously vaccinated than the current vaccination certificates, although the specific assays used for this purpose would need additional regulatory oversight.

## Future outlook

As viral adaptation to humans continues apace, it is possible that herd immunity to SARS‐CoV‐2 will not be achieved, even after vaccination, although this remains to be seen. This necessitates the ongoing urgent need for vaccination and the characterization of the molecular response to infection/vaccination over longer timeframes, as well as knowledge about how responses change with regard to different viral variants—to protect those most vulnerable. Indeed, if SARS‐CoV‐2 is not eradicated, it may turn out to be the case that children born today, unless vaccinated, contract SARS‐CoV‐2 alongside other respiratory infections of modernity, such as flu, rhinoviruses and endemic CoVs. By the time they reach adulthood, with what we know about SARS‐CoV‐2 and other infections, it holds that these children will be better equipped immunologically to face re‐infection and will accordingly suffer lower morbidity and mortality in response to infection that naïve adults and elderly people do today. Alternatively, new mutations in S could increase re‐infection, transmission and disease severity, even in the young, arguing for discussion of vaccination in all age groups, as equitably as possible. Thus, many questions remain about this new human infection. Despite the global challenges posed by SARS‐CoV‐2, the pandemic provides an unrivalled opportunity to learn about viruses and the human immune system at the population level and improve pandemic preparedness through global cooperation.

## Conflict to interest

The authors declare that there is no conflict of interest that could be perceived as prejudicing the impartiality of the research reported.
